# Brain Metastases With Malignant Peritoneal Mesothelioma: Never Reported Before

**DOI:** 10.7759/cureus.43744

**Published:** 2023-08-19

**Authors:** Darshan Lal, Merissa Misiura, Waqas Adeel, Rizwan Tariq

**Affiliations:** 1 Hematology and Medical Oncology, Lehigh Valley Health Network, Allentown, USA; 2 Internal Medicine, Lehigh Valley Health Network, Allentown, USA

**Keywords:** treatment choices, treatment, malignant pleural mesothelioma (mpm), mesothelioma, cancer immunotherapy, brain met, malignant peritoneal mesothelioma

## Abstract

Malignant mesothelioma is a very rare diagnosis. Malignant mesotheliomas arise from surface linings of pleura, peritoneal cavity, or tunica vaginalis and pericardium with pleural malignant mesotheliomas being the most common. The incidence of brain metastases has been very low with malignant pleural mesotheliomas, but to date, there have not been any cases reported of brain metastasis with malignant peritoneal mesotheliomas. We present a patient diagnosed with malignant peritoneal mesothelioma and was successfully treated with immunotherapy for over two years but later presented with brain metastases. Although the patient had a surgical resection followed by brain radiation, he died three months after his diagnosis of brain metastases. Immunotherapy has revolutionized the treatment of malignant mesothelioma, and patients are living longer than before. We present this patient to increase awareness of brain metastases with malignant peritoneal mesothelioma. This case also highlights that we need to investigate different treatment options for brain metastases in patients with malignant mesothelioma as conventional treatment options like surgical resection and brain radiation are not very effective.

## Introduction

Malignant mesothelioma is a rare disease. Of all mesothelioma cases, 85% originate from the pleura and only 15% originate from the peritoneum [1]. As it is a rare diagnosis with overlapping histological features, the accurate diagnosis remains a challenge. Subtypes of malignant mesothelioma include epithelioid, sarcomatoid, and biphasic/mixed. The latter two subtypes have a significantly worse prognosis. Distant metastases are not uncommon due to malignant pleural and peritoneal mesothelioma, but brain metastases are exceedingly rare. The incidence of brain metastases with malignant pleural mesothelioma has been estimated to be 2.7% [1]. We did not find any cases reported so far of brain metastases related to diffuse peritoneal mesothelioma. We present a case of a middle-aged male patient with malignant peritoneal mesothelioma who was later diagnosed with brain metastases.

## Case presentation

**Figure 1:**
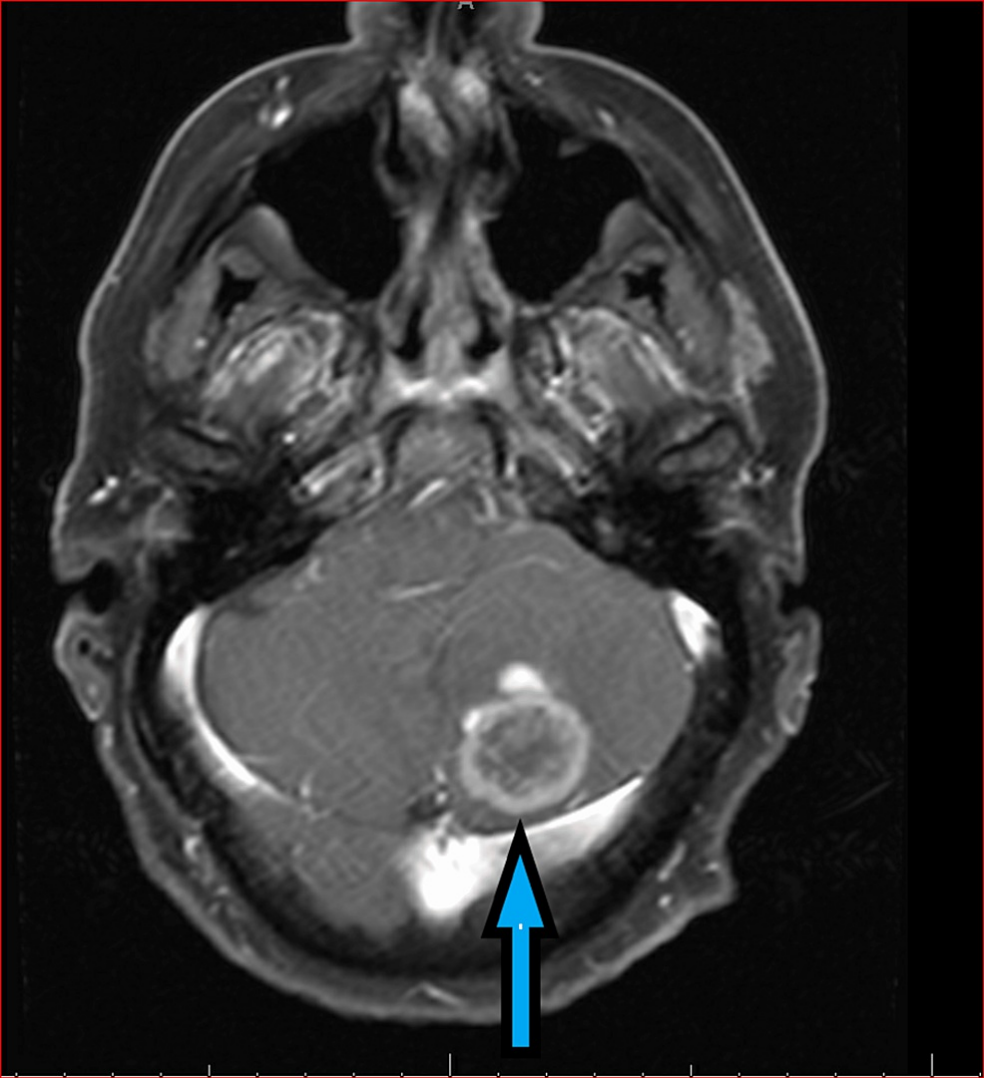
MRI brain image The image shows a peripherally enhancing mass in the left cerebellum measuring 3.2 x 2.9 x 2.9 cm.

A 59-year-old man presented with abdominal pain and weight loss in May 2020. He was found to have a Liver mass measuring 11.5 x 8 cm on the CT abdomen. Ultrasound-guided liver mass biopsy revealed sarcomatoid carcinoma. It was strongly positive for AE1/AE3, GATA3 and CK7 but negative for calretinin, S100, CK20, TTF-1, CK20, p40, CA-19-9, CDX2. The immunostains were favoring sarcomatoid malignancy but were non-specific. Given an overall negative positron emission tomography (PET) scan except for a large perihepatic/hepatic soft tissue mass with substantial metabolic activity, the diagnosis of localized cholangiocarcinoma with sarcomatoid features was considered. In preparation for surgical resection of the mass, he underwent diagnostic laparoscopy which revealed omental and peritoneal carcinomatosis. Biopsies raised the suspicion of malignant peritoneal mesothelioma. He underwent partial hepatectomy for optimal debulking and pathology confirmed malignant peritoneal mesothelioma given its positivity for D2-40. Hyperthermic intraperitoneal chemotherapy (HIPEC) was considered but was not offered due to unclear diagnosis at the time of surgical resection. Given lymphovascular invasion and gross residual disease, systemic therapy with cisplatin and pemetrexed was initiated in July 2020. After three cycles of chemotherapy, his cancer progressed locally, and in September 2020 ipilimumab and nivolumab were initiated based on CheckMate-743 clinical trial. He received nivolumab (3 mg/kg intravenously once every two weeks) plus ipilimumab (1 mg/kg intravenously once every six weeks). He had an excellent response to treatment. In June 2022, he was found to have worsening peritoneal implants and he underwent complete surgical resection which was followed by a single agent Nivolumab. Unfortunately, in Jan 2023, he presented with acute headaches and was found to have a left cerebellar mass (Figure 1) confirming malignant mesothelioma on pathology. He underwent brain radiation after surgical resection but continued to have progression in the brain with multiple new brain metastases and was transitioned to hospice and died three months later.

## Discussion

Malignant mesotheliomas are very aggressive tumors. They originate from serosal surfaces such as the pleura, peritoneal, pericardium, etc. The histological subtype determines the aggressiveness of the tumor with sarcomatoid being the most aggressive [2]. Malignant mesotheliomas most commonly invade or progress locally but hematogenous metastases are not uncommon. CNS metastases are very rare [3]. Miller et al. performed pooled analysis and systemic review of more than 700 patients with diffuse malignant mesothelioma and found the prevalence of CNS metastases to be 2.7% [4]. Most of the cases were related to sarcomatoid or mixed variant but none were reported with peritoneal mesothelioma. Our literature search did not find CNS metastasis reported with peritoneal mesothelioma. Here we presented a case of peritoneal mesothelioma which later metastasized to the brain. There is no consensus on the treatment of brain metastases in mesothelioma patients. Treatment strategies have been heterogenous and overall prognosis after CNS metastases remains poor [5]. The retrospective review by Martinez et al. found median overall survival after brain metastases at 83-183 days [6]. As immunotherapy has improved the outcomes of malignant mesothelioma patients, we are seeing more patients living longer with malignant mesothelioma. Our patient lived almost three years with this diagnosis. We feel that there is an increased need to recognize CNS metastasis early. Conventional surgical resection and radiation therapy seem to have very limited efficacy as most patients die within three-six months of brain metastases diagnosis.

## Conclusions

Brain metastases with malignant pleural mesothelioma are very rare. No cases have been reported of brain metastases with malignant peritoneal mesothelioma. With the use of immunotherapy, patients with malignant mesotheliomas are living longer. Unfortunately, the median survival of patients with brain metastases due to malignant mesotheliomas is about three months. This case highlights the importance of recognizing brain metastases early with malignant peritoneal mesothelioma and looking for different treatment options than conventional surgical resection and brain radiation.
